# Efficacy and Safety of Immune Checkpoint Inhibitors in Patients with Cancer and Hepatitis B or C: A Systematic Review and Meta-Analysis

**DOI:** 10.1155/2023/2525903

**Published:** 2023-01-07

**Authors:** Huijing Dong, Chongxiang Xue, Yumin Zheng, Xu Zhang, Zixin Hu, Xingyu Lu, Yixuan Yu, Jia Li, Kexin Tan, Huijuan Cui

**Affiliations:** ^1^Beijing University of Chinese Medicine, Beijing, China; ^2^Department of Integrative Oncology, China-Japan Friendship Hospital, Beijing, China

## Abstract

**Background:**

Immune checkpoint inhibitors (ICIs) have changed the situation of tumor therapy in recent years. However, for security reasons, those special populations are often excluded from clinical trials, such as infected hepatitis B or hepatitis C patients. ICIs are systematically reviewed and meta-analyzed for the first time in patients infected with hepatitis B or C in this paper.

**Methods:**

The relevant studies were searched in PubMed, EMBASE, Cochrane Library, and Web of Science until October 2022. Trials and observational studies meeting the inclusion criteria were included. The outcomes included the effectiveness of ICIs in patients with HBC/HCV (ORR, DCR, mOS, and mPFS), the incidence of adverse reactions, high-grade adverse reactions, and abnormal liver enzymes. At the same time, these indexes were compared with those of uninfected patients.

**Results:**

A total of 2,625 patients were enrolled, involving 1,179 patients with hepatitis (HBV or HCV). We found that ICIs showed higher ORR (25.80% vs. 18.10%) and DCR (66.22% vs. 58.74%) in patients with hepatitis B/C than those without infection. In terms of survival time, patients with hepatitis virus infection showed longer mOS (15.44 m vs. 13.30 m) but shorter mPFS (4.94 m vs. 5.01 m) than uninfected patients. As for safety data, patients with hepatitis showed a lower incidence of all-grade irAEs (68.02% vs. 70.43%) than uninfected patients, while that of 3-4 irAEs (21.27% vs. 21.79%) was similar in the two groups. However, hepatic dysfunction was more common and serious in hepatitis patients. Four HBVr and no HCVr were observed.

**Conclusion:**

According to this meta-analysis, ICIs are effective and safe for patients with hepatitis B or C, but basic liver enzymes have to be evaluated before treatment to avoid liver adverse events.

## 1. Introduction

Over the past few years, the immune checkpoint inhibitors (ICIs) that target programmed cell death receptor-1 (PD-1), programmed cell death-ligand 1 (PDL1), or cytotoxic T-lymphocyte-associated protein 4 (CTLA-4) have shown encouraging efficacy in a variety of tumor types such as lung cancer, melanoma, and liver cancer [1]. Although ICIs have presented a revolutionary alternative therapeutic approach for patients with tumor, its adverse reactions also pose a threat to them.

Hepatitis C virus (HCV) and hepatitis B virus (HBV) are the main causes of chronic liver disease worldwide and are the leading causes of liver cancer and overall mortality globally [2, 3]. The total infection prevalence of HBV worldwide has risen to 3.9%, which means that at least 292 million people suffer from HBV [3]. As for HCV, about 71 million people worldwide are chronically infected with the hepatitis C virus [2]. Regardless of the fact that there are not a small number of people with hepatitis, these patients are often excluded from ICIs clinical trials for the associated theoretical risk of hyperimmune response or causing hepatitis B/C reactivation. Taking into account this concern, there is limited evidence of the safety and efficacy of ICIs in patients with viral hepatitis.

The Checkmate 040 study showed that in patients with advanced hepatocellular carcinoma, the disease control and response rates of hepatitis-infected and uninfected patients were similar and hepatitis patients did not show a higher tendency of adverse reactions [4]. A retrospective cohort study found that 5.3% of patients had HBV reactivation, which is concerning and cannot be ignored [5].

Therefore, it is crucial to conduct a comprehensive evaluation of ICIs' efficacy and safety in patients with hepatitis B/C for clinical decision-making. Previously published studies are usually retrospective and observational, with only a small number of intervention studies, so we conduct a meta-analysis to combine their results to obtain convincing evidence.

## 2. Methods

### 2.1. Search Strategy

The Preferred Reporting Items for Systematic Reviews and Meta-Analyses (PRISMA) statement was followed during this meta-analysis, and it was registered in the International Prospective Register of Systematic Reviews (PROSPERO) with the number CRD42022341247.

### 2.2. Study Selection

Searches were conducted in PubMed, EMBASE, the Cochrane Library, and Web of Science until October 2022, for studies pertaining to ICIs in tumor patients with hepatitis B or C. The search terms utilized keywords and Medical Subject Headings (MeSH) terms to define conditions such as ICIs, hepatitis B, and hepatitis C. Specific retrieval strategies are presented in Appendix.

Literature screening was carried out by two authors (HJD and CXX) independently by reading the title, abstract, and full text to select the studies eligible for inclusion that met the following inclusion criteria: (1) the type is intervention trial (randomized or nonrandomized and controlled or noncontrolled) or observational study (cohort studies, case-control studies, or case series with more than 5 hepatitis, and prospective or retrospective), (2) participants were treated with ICIs, either alone or in combination with other treatments, (3) the study reported the efficacy of ICIs in tumor patients with hepatitis B or C, with or without safety outcomes, and (4) the study was published in English.

If an update of the same population data was given, the latest literature would be selected. If there was any disagreement during the literature screening, it would be decided after a full discussion with the third researcher (HJC).

### 2.3. Data Extraction

Two authors (HJD and CXX) extracted the data independently. Incongruities would be resolved by discussions with the third author (HJC). Following are the characteristics of the extracted data in the included studies: authors, year of publication, country, study types, carcinoma, type of hepatitis, ICIs, ICIs types, number of patients, mean age, effective outcomes (mOS, mPFS, ORR, DCR), and security outcome (incidence of adverse reactions). All data were recorded in the table.

### 2.4. Assessment of Study Quality and Publication Bias

Three tools were used to adjust for different types of studies in this meta-analysis. The Cochrane risk-of-bias tool (RoB 2.0) was used for randomized controlled trials, while Risk Of Bias In Non-randomised Studies of Interventions (ROBINS I) was used for nonrandomized intervention studies. Evaluation of observational studies was done using Strengthening the Reporting of Observational Studies in Epidemiology (STROBE). In addition, potential publication bias was assessed by Begg's test.

### 2.5. Data Analysis

All statistical analyses were conducted using STATA software (version 14.10). A value of *P* < 0.05 was considered statistically significant. Meta-analysis of rates was carried out with Freeman−Tukey double arcsine transformation (metaprop command, ftt option). In order to assess statistical heterogeneity, the *I*^2^ statistic was used. Subsequently, considering the heterogeneity of research design and study types, the meta-analysis was mainly based on a random-effect model. The subgroup analysis was based primarily on the location of the tumor.

## 3. Results

### 3.1. Study Selection

**Figure 1:**
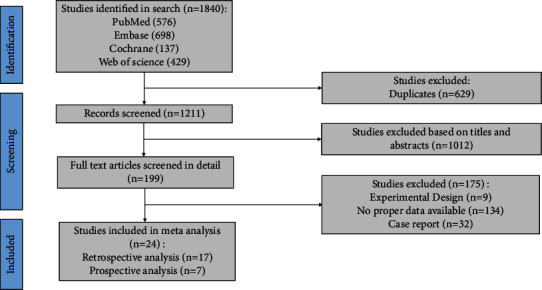
Flow diagram of search and selection.

As a result of the retrieval strategy, we identified 1840 records, which were subsequently reduced to 1211 after removing duplicate records. Using the title and abstract to assess eligibility, 1012 studies were excluded. Reading 199 studies in their entirety, 24 articles [4, 6–28] were deemed to be eligible for inclusion, including 7 prospective studies and 17 retrospective studies. Finally, 2,625 patients were enrolled, involving 1,179 patients with hepatitis (HBV or HCV). In Figure 1, one can see a detailed description of the retrieval process.

### 3.2. Study Characteristics

The baseline characteristics of 24 studies are summarized in Supplementary Table S1 and Supplementary Table S2. The inclusion criteria of hepatitis patients in each study are detailed in Supplementary Table S3. Among all the studies included, the efficacy of ICIs in patients with HBV or HCV was evaluated and safety was evaluated in 13 studies. Among 1,179 patients with hepatitis, 11 studies included patients with HBV, 2 studies included patients with HCV, and 11 studies included patients with HBV or HCV. In ICIs, the vast majority was anti-PD-1 or anti-PD-L1 (19), followed by anti-CTLA-4 (4). 1 study was anti-PD-1 combined with anti-CTLA-4, and 1 study did not specify the type of ICIs. The categories of tumors included the following: liver (15), lung (8), melanoma (4), kidney (3), stomach (2), colorectum (1), biliary (1), esophagus (1), head and neck tumor (1), glioblastoma (1), and urothelium (1).

### 3.3. Clinical Efficacy Response

#### 3.3.1. ORR

**Figure 2:**
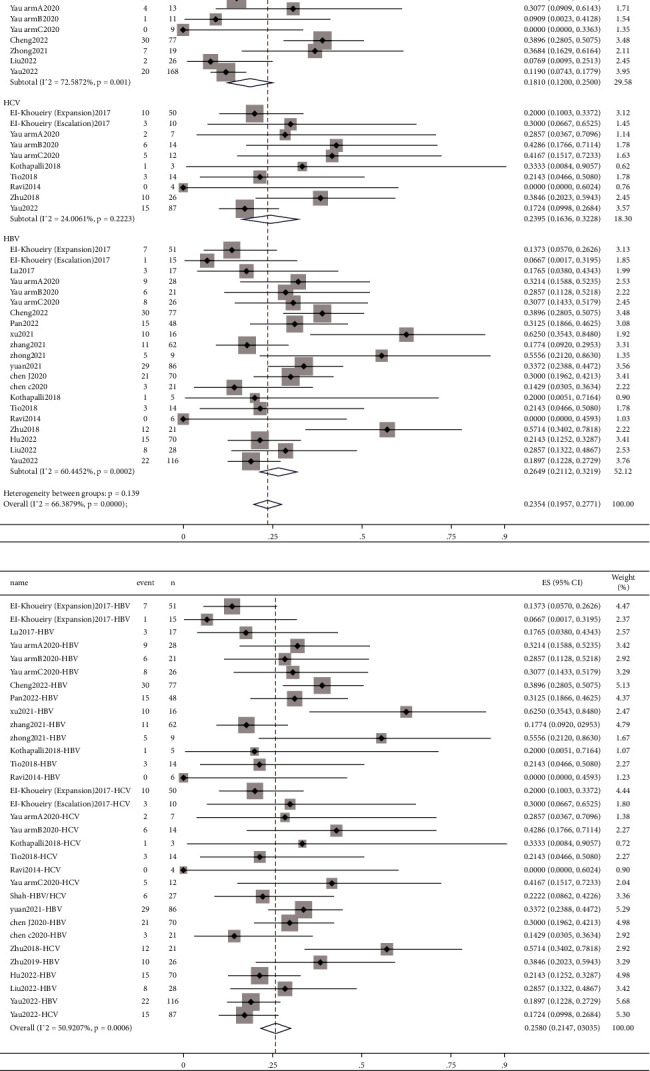
Forest plots depicting pooled ORR of (a) uninfected patients and patients with HCV and HBV and (b) patients with hepatitis.

A total of 21 studies reported ORR data [4, 6–9, 11–14, 16, 18–28], involving 815 uninfected patients and 1,061 hepatitis patients. This meta-analysis showed (Figure 2) that the pooled ORR of uninfected patients, HCV patients, and HBV patients was 18.10% (95% CI: 12.00%–25.00%), 23.95% (95% CI: 16.36%–32.28%), and 26.49% (95% CI: 21.12%–32.19%), respectively. The pooled ORR of 1,061 hepatitis patients was 25.80% (95% CI: 21.47%–30.35%). In subgroup analysis among hepatitis patients, the liver group showed higher pooled value (25.97%, 95% CI: 21.36%–30.83%) than other tumors group (25.14%, 95% CI: 15.34%–36.12%) (Supplementary Figure 1). If one study included both liver tumors and other tumors, it would be classified into other tumors group in the subgroup analysis, which was also the same in other analysis..

#### 3.3.2. DCR

**Figure 3:**
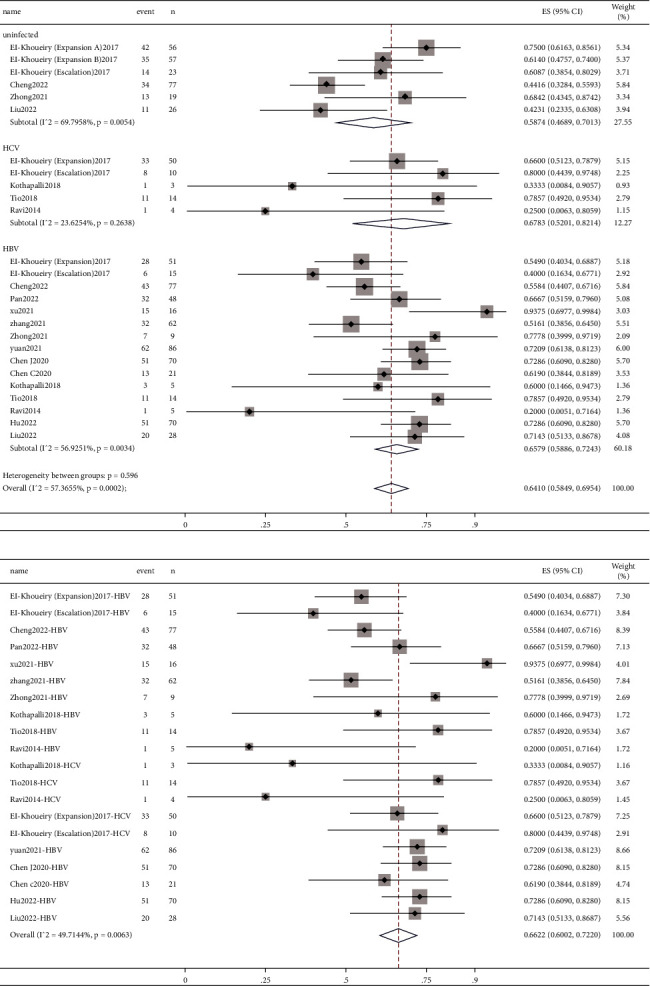
Forest plots depicting pooled DCR of (a) uninfected patients and patients with HCV and HBV and (b) patients with hepatitis.

Fourteen studies included DCR parameters [4, 6–8, 12–14, 16, 20–23, 26, 27]. The pooled DCR of uninfected patients and HCV and HBV patients was 58.74% (95% CI: 46.89%–70.13%), 67.83% (95% CI: 52.01%–82.14%), and 65.79% (95% CI: 58.86%–72.43%). After the merger of 658 patients with hepatitis, the pooled value was 66.22% (95% CI: 60.02%–72.20%). The forest plot is given in Figure 3. According to the subgroup analysis based on the categories of tumors among hepatitis patients, the pooled value of the liver group was 67.67% (95% CI: 62.30%–72.82%), which was higher than other tumors group (64.10%, 95% CI: 49.96%–77.28%) (Supplementary Figure 1).

#### 3.3.3. mPFS

**Figure 4:**
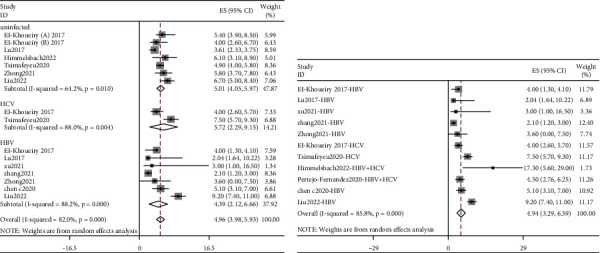
Forest plots depicting pooled mPFS of (a) uninfected patients and patients with HCV and HBV and (b) patients with hepatitis.

Median progression-free survival (mPFS) was published by 11 studies [8, 9, 12, 15, 17, 19, 20, 22–24, 27]. The pooled mPFS (Figure 4) was 5.01 months (95% CI: 4.05–5.97) for uninfected patients, 5.72 months (95% CI: 2.29–9.15) for patients with HCV, and 4.39 months (95% CI: 2.12–6.66) for patients with HBV. For all patients with hepatitis, the pooled value was 4.94 months (95% CI: 3.29–6.60). In addition, we calculated the mean of mPFS in these four sets of data, which was 4.83 months (uninfected), 5.75 months (HCV), 4.15 months (HBV), and 5.96 months (hepatitis). The pooled value of liver tumors (5.98 months, 95% CI: 3.55–8.42) was longer than that of the other tumors group (4.02 months, 95% CI: 1.73–6.31) of patients with hepatitis (Supplementary Figure 1).

#### 3.3.4. mOS

**Figure 5:**
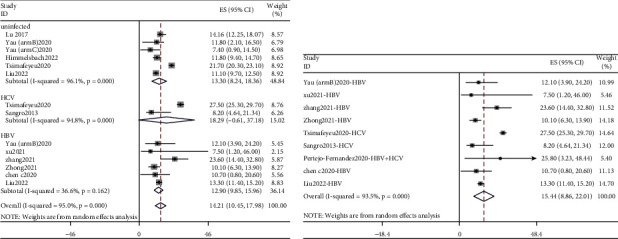
Forest plots depicting pooled mOS of (a) uninfected patients and patients with HCV and HBV and (b) patients with hepatitis.

Median overall survival (mOS) was published by 12 studies [8–12, 15, 17, 19, 20, 22, 23, 27]. According to the meta-analysis, the pooled mOS was 13.30 months (95% CI: 8.24–18.36), 18.29 months (95% CI: −0.61–37.18), 12.90 months (95% CI: 9.85–15.96), and 15.44 months (95% CI: 8.86–22.01) for patients who were uninfected and those who had HCV, HBV, and hepatitis (Figure 5). The pooled mOS of liver tumors was 12.94 months (95% CI: 11.15–14.74), which is shorter than that of the other tumors group (19.37 m, 95% CI: 8.37–30.38) (Supplementary Figure 1). We also calculated the mean of mOS, with values 14.17 m (uninfected), 19.94 m (HCV), 14.67 m (HBV), and 17.17 m (hepatitis), respectively.

### 3.4. Adverse Events

**Figure 6:**
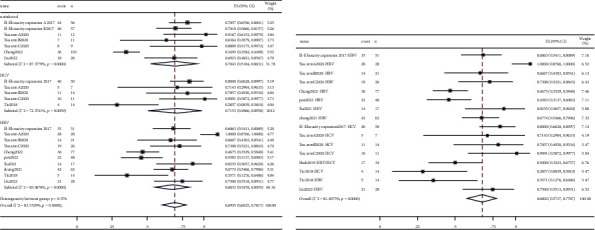
Forest plots depicting the pooled value of all-grade irAEs of (a) uninfected patients and patients with HCV and HBV and (b) patients with hepatitis.

**Figure 7:**
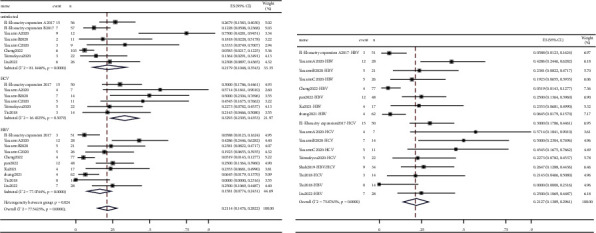
Forest plots depicting the pooled value of grade 3-4 irAEs of (a) uninfected patients and patients with HCV and HBV and (b) patients with hepatitis.

The incidence of immune-related adverse events (irAEs) in hepatitis patients and uninfected patients was further combined. A total of 13 studies reported irAEs [4, 7, 8, 10–12, 14, 16, 18–20, 22, 27]. irAEs were classified into all grades and grades 3-4. The highest pooled value of all-grade irAEs was HCV patients (71.53%, 95% CI: 49.66%–89.58%), followed by uninfected patients (70.43%, 95% CI: 51.84%–86.31%), HBV patients (68.33%, 95% CI: 54.78%–80.55%), and hepatitis patients (68.02%, 95% CI: 57.37%–77.87%). Forest plots are presented in Figure 6. The pooled value of the incidence of grade 3-4 irAEs was, respectively, 21.79% (95% CI: 10.48%–35.43%), 32.93% (95% CI: 23.05%–43.53%), 15.18% (95% CI: 7.74%–24.31%), and 21.27% (95% CI: 13.89%–29.61%) in uninfected patients and those who had HCV, HBV, and hepatitis (Figure 7).

Among hepatitis patients, the pooled incidence of all-grade irAEs in the liver group (76.67%, 95% CI: 64.06%–87.45%) was higher than in other tumors group (53.34%, 95% CI: 39.35%–67.08%) (Supplementary Figure 2). The pooled value of grade 3-4 irAEs showed similar results: the liver group value was 28.19% (95% CI: 18.48%–38.93%), and the other tumors group value was 12.36% (95% CI: 4.92%–22.04%) (Supplementary Figure 2).

We further analyzed glutamic pyruvic transaminase (ALT) and glutamic oxaloacetic transaminase (AST). Patients with HCV show a higher pooled incidence rate of elevated liver enzymes, both in all-grades and grade 3-4 irAEs. Detailed data are provided in Table 1 and Supplementary Figure 3.

### 3.5. Subgroup Analysis

**Figure 8:**
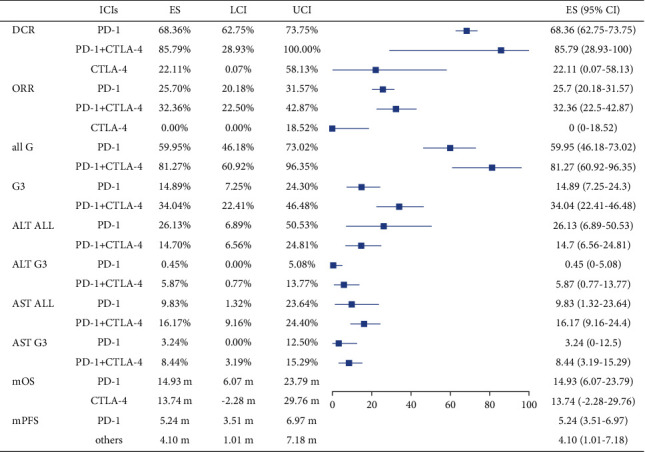
The subgroup analysis of ICI types.

In addition to the location of the tumor, we carried out a subgroup analysis of ICI types. The results suggested that the combination of ICIs showed higher ORR and DCR. At the same time, the incidence of irAEs for patients receiving combined therapy of anti-PD-1 and anti-CTLA-4 drugs was higher than that of a single drug (81.27% vs. 59.95%) and the incidence of high-grade irAEs was also higher (34.04% vs. 14.89%). Detailed results are provided in Figure 8. The forest plots are shown in Supplementary Figure 4.

### 3.6. Quality Assessment and Publication Bias

**Figure 9:**
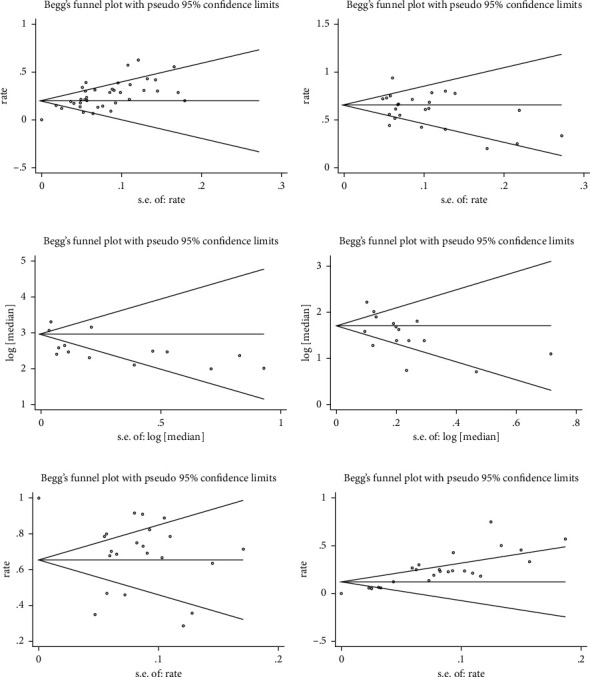
Begg's funnel plots. (a) ORR. (b) DCR. (c) mOS. (d) mPFS. (e) All-grade irAEs. (f) Grade 3-4 irAEs.

We evaluated 2 randomized studies with ROB 2.0, 5 nonrandomized intervention studies with ROBINS-I, and 17 observational studies with STROBE. Only one study showed high risk, while others showed low to medium risk (Supplementary Table S4). There was a publication bias in ORR and grade 3-4 irAEs calculated by Begg's test and funnel plots, and others did not show publication bias (Figure 9). Sensitivity analysis indicated that the results were stable, and the detailed results are reported in Supplementary Figure 5.

## 4. Discussion

There has been no systematic review or meta-analysis that evaluated ICIs' efficacy and safety in patients with hepatitis B or C based on the available studies. In our study, we found that ICIs showed higher ORR (25.80% vs. 18.10%) and DCR (66.22% vs. 58.74%) in patients with hepatitis B/C than those without infection. In terms of survival time, patients with hepatitis virus infection showed longer mOS (15.44 m vs. 13.30 m) but shorter mPFS (4.94 m vs. 5.01 m) than uninfected patients. As for safety data, patients with hepatitis showed a lower incidence of all-grade irAEs (68.02% vs. 70.43%) than uninfected patients, while that of 3-4 irAEs (21.27% vs. 21.79%) was similar in the two groups. However, hepatic dysfunction wasmore common and serious in hepatitis patients.

Immune escape of tumor cells is mediated by the PD-1/PD-L1 axis and CTLA-4. PD-1 is a negative regulatory cell surface receptor and an important regulator of the adaptive immune response, which is expressed in T cells, B cells, macrophages, and so on. Two ligands of PD-1, PD-L1 (B7-H1; CD274) and PD-L2 (B7-DC; CD273), can downregulate the effector function of T cells by binding to PD-1 [29]. CTLA-4 is a member of a family of immunoglobulin-related receptors, which is predominantly found in intracellular vesicles in FoxP3 regulatory T cells (Tregs) or activated conventional T cells. CTLA-4 is homologous to T-cell costimulatory protein CD28 and shares two ligands, namely, CD80 and CD86. The interaction between ligands and CTLA-4 is helpful to inhibit T-cell response [30]. ICIs can restore T-cell function by blocking the binding of PD-1 or CTLA-4 to ligands, thus achieving the purpose of tumor therapy. However, in patients with hepatitis B/C, this situation becomes complicated.

There has been evidence that PD-1 was significantly overexpressed on total and HCV-specific CD8 cytotoxic T lymphocytes (CTLs) in the liver and peripheral blood of patients with persistent HCV infection [31]. Similarly, HBV-specific T cells in the peripheral blood of patients with chronic HBV infection also express high levels of PD-1 [32]. In addition to PD-1, the upregulation of CTLA-4 on virus-specific T cells from chronic HBV and HCV was likewise repeatedly observed [33]. Under the action of negative costimulatory molecules such as CTLA-4 and PD-1, specific T-cell dysfunction occurs in patients with persistent hepatitis B or C infection. Therefore, while blocking this process to inhibit tumors, ICIs may also reverse T-cell depletion and play an antiviral effect, which has been observed in some clinical studies. El-Khoueiry [8] observed a transient decrease of hepatitis C virus RNA in some patients infected with HCV, showing limited antiviral activity of nivolumab. Sangro [10] identified a decrease in viral load after treatment with tremelimumab and a transient complete viral response in three patients during follow-up. According to a recent study, this antiviral effect may be related to ICIs' effects on Tregs, which plays an important role in HBV and HCV patients [34].

In addition, Han found that serum-soluble PD-L1 (sPD-L1) levels in patients with HBV-related hepatocellular carcinoma (HCC) was markedly increased, which was positively correlated with the expression of PD-L1 in tumor tissues [35]. The upregulation of PD-L1 was also observed in patients with HCV infection [36]. In view of the fact that the expression level of PD-L1 in tumor tissues has become a biomarker for predicting the efficacy of immunotherapy [37], it can be speculated that the high ORR and DCR of ICIs in patients with HCV/HBV are related to the high expression of PD-L1.

However, ICIs may also weaken the ability of T cells to inhibit viral hepatitis, resulting in HBV/HCV reactivation [6]. The incidence of HBV reactivation (HBVr) and HCV reactivation (HCVr) induced by immunotherapy is not clear. Among the 878 hepatitis patients we included, 4 HBVr and no HCVr were observed [16, 22, 26], so we thought that the probability of hepatitis reactivation caused by ICIs was relatively low. In addition to HBVr and HCVr, immune-mediated hepatotoxicity constitutes one of the reasons why researchers exclude clinical trials because hepatitis patients are often accompanied by baseline damage of liver function. Immune-mediated hepatic dysfunction was found to be more common and severe in patients with hepatitis than in patients without infection, according to our study. In HBV patients, this is more prevalent than in HCV patients. The reason for this might be that patients with HCV have a higher risk of suffering from liver damage (micronodular cirrhosis, lymphoid aggregates, damage to the bile ducts, etc.) than those with HBV [38]. Meanwhile, in the subgroup analysis, the incidence of immune-mediated hepatic dysfunction was higher in those given the combination of anti-PD-1 and anti-CTLA-4, both in all grades and high grades (except the incidence of ALT increases in all grades).

There are certain limitations that originated from the finite number of studies present in this meta-analysis. The literature included in this study is dominated by observational studies. Therefore, the interpretation still needs to be further verified by larger samples and randomized clinical trials. In addition, the majority of patients in related studies were diagnosed with liver cancer, possibly because HBV/HCV and liver cancer are closely related, which may lead to a certain bias. In addition, due to the lack of analysis of viral hepatitis stages in many studies, there was no subgroup analysis of this factor.

## 5. Conclusion

According to this meta-analysis, ICIs in patients with hepatitis B or C are effective and safe, but the baseline of liver enzyme should be evaluated before use, especially when multiple ICIs are used in combination. Besides, the infectious disease physician should be invited to evaluate and follow up the patients.

## Figures and Tables

**Table 1:** The pooled incidence rate of AST and ALT abnormalities.

Characteristics		Incidence (%)	95% CI	*P*	*I* ^2^ (%)
*AST increase*					
All grades	Uninfected	14.17	5.18%–25.86%	0.086	51.01
	HCV	24.20	4.79%–50.46%	<0.001	83.11
	HBV	12.26	5.61%–20.71%	0.026	60.63
	Hepatitis	16.93	8.32%–27.45%	<0.001	76.97
Grades 3-4	Uninfected	3.16	0.01%–9.43%	0.211	31.57
	HCV	17.37	5.51%–32.96%	0.043	59.43
	HBV	3.09	0.34%–7.56%	0.121	42.57
	Hepatitis	7.69	2.30%–15.13%	<0.001	71.08

*ALT increase*					
All grades	Uninfected	8.56	1.49%–19.19%	0.096	52.79
	HCV	36.07	11.45%–64.51%	<0.001	79.99
	HBV	13.07	5.62%–22.48%	0.041	56.84
	Hepatitis	21.27	10.91%–33.49%	0.210	33.65
Grades 3-4	Uninfected	2.25	0.00%–8.19%	0.293	19.17
	HCV	9.39	2.19%–19.43%	0.409	1.10
	HBV	0.95	0.00%–3.66%	0.044	46.65
	Hepatitis	3.48	0.37%–8.46%	0.210	33.65

## Data Availability

Detailed information about the original contributions to the study is included within the article/Supplementary Materials. Further data are available from the corresponding author upon request.

## Supplementary Materials

Supplementary Materials The following supporting information can be obtained from Supplementary Materials: Appendix: search strategy; Table S1: basic characteristics of the included studies; Table S2: immune-related adverse reactions of the included studies; Table S3: HCV/HBV inclusion criteria of the included studies; Table S4: risk of bias assessment; Figure S1: subgroup analysis of ORR, DCR, mPFS, and mOS; Figure S2: subgroup analysis of all-grade irAEs and grade 3-4 irAEs; Figure S3: the pooled incidence rate of AST, ALT abnormalities; Figure S4: the subgroup analysis of ICIs; Figure S5: the sensitivity analysis.
