# Supplementary material for: Efficacy and Safety of Immune Checkpoint Inhibitors in Patients with Cancer and Hepatitis B or C: A Systematic Review and Meta-Analysis
Source: J Oncol. 2023 Jan 7;2023:2525903. doi: 10.1155/2023/2525903 (PMC9840556; doi:10.1155/2023/2525903)
Supplement: Supplementary Materials — The following supporting information can be obtained from Supplementary Materials: Appendix: search strategy; Table S1: basic characteristics of the included studies; Table S2: immune-related adverse reactions of the included studies; Table S3: HCV/HBV inclusion criteria of the included studies; Table S4: risk of bias assessment; Figure S1: subgroup analysis of ORR, DCR, mPFS, and mOS; Figure S2: subgroup analysis of all-grade irAEs and grade 3-4 irAEs; Figure S3: the pooled incidence rate of AST, ALT abnormalities; Figure S4: the subgroup analysis of ICIs; Figure S5: the sensitivity analysis. [file 2525903.f1.docx]

**Appendix. Search strategy.**

Cochrane 137 results

#1 (Camrelizumab:ab) OR (Nivolumab:ab) OR (Pembrolizumab:ab) OR (Toripalimab:ab) OR (Tislelizumab:ab) OR (Sintilimab:ab) OR (Durvalumab:ab) OR (Atezolizumab:ab) OR (Avelumab:ab) OR ('PD-?1':ab) OR ('programmed death receptor 1':ab) OR (‘programmed cell death-Ligand 1':ab) OR (Ipilimumab:ab) OR (Tremelimumab:ab) OR ('CTLA-4':ab) OR ('cytotoxic T-lymphocyte-associated protein 4':ab)

#2('hepatitis B':ab) OR ('hepatitis C':ab)

#3 #1AND#2

Embase 698 results

#1 (Camrelizumab:ab) OR (Nivolumab:ab) OR (Pembrolizumab:ab) OR (Toripalimab:ab) OR (Tislelizumab:ab) OR (Sintilimab:ab) OR (Durvalumab:ab) OR (Atezolizumab:ab) OR (Avelumab:ab) OR ('PD-?1':ab) OR ('programmed death receptor 1':ab) OR (‘programmed cell death-Ligand 1':ab) OR (Ipilimumab:ab) OR (Tremelimumab:ab) OR ('CTLA-4':ab) OR ('cytotoxic T-lymphocyte-associated protein 4':ab)

#2('hepatitis B':ab) OR ('hepatitis C':ab)

#3 #1AND#2

Pubmed 576 results

#1((((((((((((((((Camrelizumab[Title/Abstract]) OR (Nivolumab[Title/Abstract])) OR (Pembrolizumab[Title/Abstract])) OR (Toripalimab[Title/Abstract])) OR (Tislelizumab[Title/Abstract])) OR (Sintilimab[Title/Abstract])) OR (Durvalumab[Title/Abstract])) OR (Atezolizumab[Title/Abstract])) OR (Avelumab[Title/Abstract])) OR (PD-?1[Title/Abstract])) OR (programmed death receptor 1[Title/Abstract])) OR (programmed cell death-Ligand 1[Title/Abstract])) OR (Ipilimumab[Title/Abstract])) OR (Tremelimumab[Title/Abstract])) OR (CTLA-4[Title/Abstract])) OR (cytotoxic T-lymphocyte-associated protein 4[Title/Abstract]))

#2 (hepatitis B[Title/Abstract]) OR (hepatitis C[Title/Abstract])

#3 #1AND#2

WOS 429 results

#1 (((((((((((((((AB=(Camrelizumab)) OR AB=(Nivolumab)) OR AB=(Pembrolizumab)) OR AB=(Toripalimab)) OR AB=(Tislelizumab)) OR AB=(Sintilimab)) OR AB=(Durvalumab)) OR AB=(Atezolizumab)) OR AB=(Avelumab)) OR AB=(PD-?1)) OR AB=(programmed death receptor 1)) OR AB=(programmed cell death-Ligand 1)) OR AB=(Ipilimumab)) OR AB=(Tremelimumab)) OR AB=(CTLA-4)) OR AB=(cytotoxic T-lymphocyte-associated protein 4)

#2 (AB=(hepatitis B)) OR AB=(hepatitis C)

#3 #1AND#2

**1. Supplementary Tables**

**1.1** **Table S1: Basic characteristics of the included studies.**

| Authors | | Year | country | Study  types | Carcinoma | HCV/HBV | ICIs | ICIs types | Number  (ALL/HCV/HBV) | Mean age |
| --- | --- | --- | --- | --- | --- | --- | --- | --- | --- | --- |
| El-Khoueiry [1] | Expansion | 2017 | USA, Spain, Hong Kong, Singapore | A^*^ | HCC | HCV or HBV | nivolumab | Anti-PD-1 | 214/50/51 | 64 |
|  | Escalation |  |  |  |  |  |  |  | 48/10/15 | 62 |
| Kelley[2] | | 2022 |  | A | biliary cancers | HCV or HBV | Pembrolizumab | Anti-PD-1 | 42/10 | 61 |
| Lu[3] | | 2017 | Asian | A | NSCLC | HBV | nivolumab | Anti-PD-1 | 400/?/17 |  |
| Sangro[4] | | 2013 | Spain | A | HCC | HCV | tremelimumab | Anti-CTLA-4 | 21/21/? | 65.2 |
| Yau [5] | armA | 2020 | Asia,Europe,North America | A | HCC | HCV or HBV | Nivolumab,ipilimumab | Anti-PD-1  + Anti-CTLA-4 | 50/7/28 | 61 |
|  | armB |  |  |  |  |  |  |  | 49/14/21 | 65 |
|  | arm C |  |  |  |  |  |  |  | 49/12/26 | 58 |
| Zhu[6] | | 2018 | Multicenter, international | A | HCC | HCV or HBV | Pembrolizumab | Anti-PD-1 | 104/26/22 | 68 |
| Cheng[7] | | 2022 | China | B^#^ | colorectal cancer | HBV |  | Anti-PD-1 | 154/?/77 | / |
| Himmelsbach[8] | | 2022 | Germany and Austria | B | HCC | HCV or HBV | Atezolizumab | Anti-PD-L1 | 66/14/9 | 65 |
| Pan[9] | | 2022 | China | B | liver cancer | HBV | Sintilimab ,camrelizumab ,  toripalimab | Anti-PD-1 | 480/?/48 | 55.96 |
| Pertejo-Fernandez[10] | | 2020 | America | B | NSCLC | HCV or HBV | ipilimumab | Anti-CTLA-4 | 19/5/16 |  |
| Shah[11] | | 2019 | America | B | HCC, NSCLC, RCC, head & neck, gastric, and SCLC | HCV or HBV |  | / | 50/22/15 | 62 |
| Tsimafeyeu[12] | | 2020 | Russian Federation, Belarus, Kazakhstan | B | RCC | HCV | Nivolumab | Anti-PD-1 | 44/22/? | 62 |
| xu[13] | | 2021 | China | B | lung cancer | HBV | Tislelizumab, Sintilimab, Nivolumab, Camrelizumab, Triprizumab, and Pembrolizumab. | Anti-PD-1 | 17/?/17 | 64 |
| zhang[14] | | 2021 | China | B | NSCLC | HBV | pembrolizumab, nivolumab, camrelizumab,atezolizumab | Anti-PD-(L)1 | 62/?/62 | 55 |
| Zhong[15] | | 2021 |  | B | liver, lung, esophageal, melanoma | HBV |  | Anti-PD-1 | 39/0/15 |  |
| yuan[16] | | 2021 | China | B | HCC | HBV | camrelizumab | Anti-PD-1 | 86/3/86 | 54.5 |
| Chen J[17] | | 2020 | China | B | HCC | HBV | toripalimab、camrelizumab 或 sintilimab | Anti-PD-1 | 70/？/70 | 53.5 |
| Chen C[18] | | 2020 | China | B | HCC | HBV | nivolumab | Anti-PD-1 | 22/?/22 | 53 |
| Kothapalli[19] | | 2018 | Australia | B | melanoma or NSCLC | HCV or HBV | Pembrolizumab, Nivolumab | Anti PD-1 | 7/3/5 | 62.9 |
| Tio[20] | | 2018 | USA | B | Melanoma,HCC,Glioblastoma, Gastric carcinoma,Urothelial carcinoma,RCC,NSCLC | HCV or HBV | Pembrolizumab, Nivolumab, ipilimumab, Atezolizumab | Anti PD-(L)1, Anti CTLA-4 | 28/14/14 |  |
| Ravi[21] | | 2014 | USA | B | melanoma | HCV or HBV | ipilimumab | Anti CTLA-4 | 9/4/5 | 57.78 |
| Hu[22] | | 2022 | China | B | HCC | HBV | \ | Anti PD-1 | 70/?/70 | 52.5 |
| Liu[23] | | 2022 | China | B | HCC | HBV | Camrelizumab | Anti PD-1 | 54/0/28 | 55.9 |
| Yau[24] | | 2022 | Asia, Australasia, Europe, and North America | A | HCC | HCV or HBV | Nivolumab | Anti PD-1 | 371/87/116 | 65 |

**Continued**

| Authors | | DCR. (%) | | | ORR. (%) | | | Median OS (m) | | | Median PFS (m) | | |
| --- | --- | --- | --- | --- | --- | --- | --- | --- | --- | --- | --- | --- | --- |
|  | | Uninfected | HCV | HBV | Uninfected | HCV | HBV | Uninfected | HCV | HBV | Uninfected | HCV | HBV |
| El-Khoueiry  [1] | Expansion | C^⁑^ : 75%  D^⁜^ : 61% | 66% | 55% | C :23%  D: 21% | 20% | 14% | D :  13.2 (8.6 to NA) | NA | NA | C:  5.4 (3.9 to 8.5)  D:  4.0 (2.6 to 6.7) | 4.0 (2.6 to 5.7) | 4.0 (1.3 to 4.1) |
|  | Escalation | 61% | 80% | 40% | 13% | 30% | 7% |  |  |  |  |  |  |
| Kelley[2] | |  |  |  | 6% | 30% | |  |  |  | 2.1 | 9.2 | |
| Lu[3] | |  |  |  | 14.9% |  | 18 % | 14.16(12.25-18.07) |  | 22.31 (10.02-NA) | 3.61 (2.33-3.75) |  | 2.04 (1.64-10.22) |
| Sangro[4] | |  |  |  |  |  |  |  | 8.2 (4.64–21.34) |  |  |  |  |
| Yau  [5] | armA |  |  |  | 31% | 29% | 32% | 22.2 (8.5-NA) | 14.9 (0.7-NA) | 22.8 (7.2-NA) |  |  |  |
|  | armB |  |  |  | 9% | 43% | 29% | 11.8 (2.1-16.5) | 16.1 (6.5-NA) | 12.1 (3.9-24.2) |  |  |  |
|  | armC |  |  |  | 0 | 42% | 31% | 7.4 (0.9-14.5) | 33.0 (3.1-NA) | 9.6 (6.0-NA) |  |  |  |
| Zhu[6] | |  |  |  |  | 39% | 57% |  |  |  |  |  |  |
| Cheng[7] | | 44% |  | 56% | 39% |  | 39% |  |  |  |  |  |  |
| Himmelsbach[8] | |  |  |  |  |  |  | 11.8 ( 9.4–14.7) | NA | | 6.1 (3.1–8.9 ) | 17.3(5.6–29 ) | |
| Pan[9] | |  |  | 66.7% |  |  | 31.3% |  |  |  |  |  |  |
| Pertejo-Fernandez  [10] | |  |  |  |  |  |  |  | 25.8 (3.23-48.44) | |  | 4.5( 2.76-6.25） | |
| Shah[11] | |  |  |  |  | 22% | |  |  |  |  |  |  |
| Tsimafeyeu  [12] | |  |  |  | 23% | 27% |  | 21.7 (20.3–23.1) | 27.5 (25.3–29.7) |  | 4.9(4-5.8) | 7.5(5.7~9.3) |  |
| xu[13] | |  |  | 93.7% |  |  | 62.5% |  |  | 7.5 (1.2 -46.0) |  |  | 3 (1-16.5) |
| zhang[14] | |  |  | 51.6% |  |  | 17.7% |  |  | 23.6 (14.4-32.8) |  |  | 2.1 (1.2–3.0) |
| Zhong[15] | | 68.4% |  | 77.8% | 36.8% |  | 55.6% | NA |  | 10.1(6.3-13.9） | 5.8(3.7-7.8） |  | 3.6(0-7.5） |
| yuan[16] | |  |  | 80.6% |  |  | 33.7% |  |  |  |  |  |  |
| Chen J[17] | |  |  | 72.9% |  |  | 30% |  |  | NA |  |  | NA |
| Chen C[18] | |  |  | 61.9% |  |  | 14.3% |  |  | 10.7 (0.8-20.6） |  |  | 5.1(3.1-7.0) |
| Kothapalli[19] | |  | 33.3% | 60% |  | 33.3% | 20% |  |  |  |  |  |  |
| Tio[20] | |  | 78.6% | 78.6% |  | 21.4% | 21.4% |  |  |  |  |  |  |
| Ravi[21] | |  | 25% | 20% |  | 0% | 0% |  |  |  |  |  |  |
| Hu[22] | |  |  | 72.9% |  |  | 21.4% |  |  |  |  |  |  |
| Liu[23] | | 42.30% |  | 71.4% | 7.7% |  | 28.6% | 11.1(9.7-12.5) |  | 13.3(11.4-15.2) | 6.7(5.0-8.4) |  | 9.2(7.4-11.0) |
| Yau[24] | |  |  |  | 12% | 17% | 19% |  |  |  |  |  |  |

A^*^, Prospective; B^#^, Retrospective; C^⁑^, sorafenib untreated or intolerant; D^⁜^, sorafenib progressor; ICIs, immune checkpoint inhibitors; ORR, objective response rate; DCR, disease control rate; CTLA-4, cytotoxic T lymphocyte-associated protein 4; PD-1, programmed cell death 1; PD-L1, programmed cell death ligand 1; HCV, hepatitis C virus; HBV, hepatitis B virus; NSCLC, non-small cell lung cancer; RCC, renal cell carcinoma; HCC, hepatocellular carcinoma; NA, not available.

**1.2** **Table S2: Immune-related adverse reactions of the included studies.**

| **Authors** |  |  | El-Khoueiry (expansion)[1] | Sangro  [4] | Yau [5] | | | Cheng  [7] | Pan  [9] | Shah  [11] | Tsimafeyeu  [12] | Xu  [13] | Zhang  [14] | Chen C  [18] | Kothapalli  [19] | Tio  [20] | Liu  [23] |
| --- | --- | --- | --- | --- | --- | --- | --- | --- | --- | --- | --- | --- | --- | --- | --- | --- | --- |
|  |  |  |  |  | armA | armB | armC |  |  |  |  |  |  |  |  |  |  |
| **Year** |  |  | 2017 | 2013 | 2020 | | | 2022 | 2022 | 2019 | 2020 | 2021 | 2021 | 2020 | 2018 | 2018 | 2022 |
| **IRAEs (%)** | **Uninfected** | Any G | A^*^:79  B^#^: 70 |  | 92 | 64 | 89 | 35 |  |  |  |  |  |  |  |  | 69.2 |
|  |  | G3-4 | A: 27  B: 12 |  | 75 | 18 | 33 | 6 |  |  | 14 |  |  |  |  |  | 23.1 |
|  | **HCV** | Any G | 80 |  | 71 | 79 | 91 | \ |  | Any 50%  G3-4:26% |  |  |  |  |  | 28.6 |  |
|  |  | G3-4 | 30 |  | 57 | 50 | 45 | \ |  |  | 23 |  |  |  |  | 21.4 |  |
|  | **HBV** | Any G | 69 |  | 100 | 67 | 73 | 47 | 45.8 |  |  | 82.4 | 67.7 |  |  | 35.7 | 75 |
|  |  | G3-4 | 6 |  | 43 | 24 | 19 | 5 | 25 |  |  | 23.5 | 6.5 |  |  | 0 | 25 |
| **AST increase (%)** | **Uninfected** | Any G | 8 |  | 33 | 9 | 11 |  |  |  |  |  |  |  |  |  | 23.1 |
|  |  | G3-4 | 4 |  | 25 | 0 | 0 |  |  |  |  |  |  |  |  |  | 3.8 |
|  | **HCV** | Any G | 12 | 70 | 14 | 21 | 9 |  |  |  |  |  |  |  |  |  |  |
|  |  | G3-4 | 10 | 45 | 14 | 14 | 9 |  |  |  |  |  |  |  |  |  |  |
|  | **HBV** | Any G | 2 |  | 14 | 24 | 15 |  |  |  |  |  | 9.7 |  |  |  | 21.4 |
|  |  | G3-4 | 0 |  | 11 | 10 | 4 |  |  |  |  |  | 1.6 |  |  |  | 3.6 |
| **ALT increase (%)** | **Uninfected** | Any G | 6 |  | 25 | 0 |  |  |  |  |  |  |  |  |  |  | 15.4 |
|  |  | G3-4 | 2 |  | 17 | 0 |  |  |  |  |  |  |  |  |  |  | 3.8 |
|  | **HCV** | Any G | 14 | 55 | 14 | 29 |  |  |  |  |  |  |  |  | 100 |  |  |
|  |  | G3-4 | 6 | 25 | 14 | 14 |  |  |  |  |  |  |  |  | 0 |  |  |
|  | **HBV** | Any G | 6 |  | 11 | 14 |  |  |  |  |  |  | 9.7 |  | 60 |  | 25 |
|  |  | G3-4 | 0 |  | 4 | 5 |  |  |  |  |  |  | 1.6 |  | 0 |  | 7.1 |
| **Rash (%)** | **Uninfected** | Any G | 14 |  | 17 | 18 | 0 |  |  |  |  |  |  |  |  |  | 11.5 |
|  |  | G3-4 | 2 |  | 8 | 9 | 0 |  |  |  |  |  |  |  |  |  | 0 |
|  | **HCV** | Any G | 18 | 65 | 14 | 29 | 36 |  |  |  |  |  |  |  |  | 14.3 |  |
|  |  | G3-4 | 0 | 5 | 0 | 7 | 0 |  |  |  |  |  |  |  |  |  |  |
|  | **HBV** | Any G | 16 |  | 36 | 24 | 15 |  | 12.5 |  |  | 5.9 | 22.6 |  |  | 7.1 | 7.1 |
|  |  | G3-4 | 0 |  | 4 | 0 | 0 |  | 0 |  |  |  | 0 |  |  |  | 3.6 |
| **Fatigue (%)** | **Uninfected** | Any G | 30 |  | 17 | 18 | 0 |  |  |  |  |  |  |  |  |  | 11.5 |
|  |  | G3-4 | 2 |  | 0 | 0 | 0 |  |  |  |  |  |  |  |  |  | 0 |
|  | **HCV** | Any G | 16 | 55 | 57 | 21 | 18 |  |  |  |  |  |  |  |  |  |  |
|  |  | G3-4 | 2 | 0 | 14 | 0 | 0 |  |  |  |  |  |  |  |  |  |  |
|  | **HBV** | Any G | 14 |  | 11 | 5 | 12 |  | 14.6 |  |  | 23.5 | 21 | 13.9 |  |  | 14.3 |
|  |  | G3-4 | 0 |  | 0 | 0 | 0 |  | 0 |  |  |  | 1.6 |  |  |  | 0 |
| **Pruritus (%)** | **Uninfected** | Any G | 16 |  | 33 | 36 | 22 |  |  |  |  |  |  |  |  |  |  |
|  |  | G3-4 | 0 |  | 17 | 0 | 0 |  |  |  |  |  |  |  |  |  |  |
|  | **HCV** | Any G | 28 |  | 14 | 21 | 18 |  |  |  |  |  |  |  |  |  |  |
|  |  | G3-4 | 2 |  | 0 | 0 | 0 |  |  |  |  |  |  |  |  |  |  |
|  | **HBV** | Any G | 25 |  | 61 | 38 | 35 |  | 4.2 |  |  | 5.9 | 6.5 |  |  |  |  |
|  |  | G3-4 | 0 |  | 0 | 0 | 0 |  | 0 |  |  |  | 0 |  |  |  |  |
| **Diarrhoea（%）** | **Uninfected** | Any G | 17 |  | 33 | 18 | 11 |  |  |  |  |  |  |  |  |  | 15.4 |
|  |  | G3-4 | 2 |  | 17 | 0 | 0 |  |  |  |  |  |  |  |  |  | 0 |
|  | **HCV** | Any G | 10 | 30 | 14 | 21 | 9 |  |  |  |  |  |  |  |  |  |  |
|  |  | G3-4 | 0 | 5 | 0 | 7 | 0 |  |  |  |  |  |  |  |  |  |  |
|  | **HBV** | Any G | 6 |  | 25 | 5 | 19 |  | 16.7 |  |  |  | 4.8 | 9.1 |  |  | 17.9 |
|  |  | G3-4 | 2 |  | 0 | 0 | 0 |  | 4.2 |  |  |  | 0 |  |  |  | 3.6 |

A^*^, sorafenib untreated or intolerant; B^#^, sorafenib progressor; Any G, any grades; G3–4, grades 3–4; HCV, hepatitis C virus; HBV, hepatitis B virus.

**1.3 Table S3: HCV/HBV inclusion criteria of the included studies.**

| **No** | **Authors** | **Year** | **HCV/HBV inclusion criteria** |
| --- | --- | --- | --- |
| 1 | El-Khoueiry[1] | 2017 | Negative for HCV RNA and HBV DNA and/or surface antigen |
| 2 | Kelley[2] | 2022 | Not mentioned |
| 3 | Lu[3] | 2017 | Not mentioned |
| 4 | Sangro[4] | 2013 | Not mentioned |
| 5 | Yau[5] | 2020 | Hepatitis B virus (HBV)–positive patients had detectable HBV surface antigen or HBV DNA and were required to be receiving antiviral therapy and have a viral load less than 100 IU/mL at screening. Hepatitis C virus (HCV)–positive patients had detectable HCV RNA or antibody and did not require antiviral therapy. |
| 6 | Zhu[6] | 2018 | Patients with chronic infections with hepatitis C virus (treated or untreated) and patients with hepatitis B virus who were treated with antiviral therapy and who had a viral load less than 100 IU /mL before receiving their first pembrolizumab. |
| 7 | Cheng[7] | 2022 | Chronic HBV infection was defined as HBsAg positive and clinical resolved HBV infection was defined as HBsAg-negative and anti-HBc-positive |
| 8 | Himmelsbach  [8] | 2022 | Not mentioned |
| 9 | Pan[9] | 2022 | HBV DNA or HBsAg seropositivity |
| 10 | Pertejo-Fernandez[10] | 2020 | A history of past (resolved infection) or chronic HBV or chronic HCV |
| 11 | Shah[11] | 2019 | Not mentioned |
| 12 | Tsimafeyeu  [12] | 2020 | Not mentioned |
| 13 | Xu[13] | 2021 | Not mentioned |
| 14 | Zhang[14] | 2021 | Serum HBsAg positive or HBsAg negative and hepatitis B core antibody (HBcAb) positive; |
| 15 | Zhong[15] | 2021 | Positive HBsAg test. |
| 16 | Yuan[16] | 2021 | Seropositive for HBsAg |
| 17 | Chen J[17] | 2020 | Chronic HBV infection |
| 18 | Chen C[18] | 2020 | Recurrent hepatitis B virus-related HCC |
| 19 | Kothapalli[19] | 2018 | Past/current HBV or HCV infection |
| 20 | Tio[20] | 2018 | Not mentioned |
| 21 | Ravi[21] | 2014 | Inactive or active |
| 22 | Hu[22] | 2022 | Seropositive for HBsAg and had received tenofovir alafenamide fumarate therapy as a regular antiviral regimen before anti-PD-1 treatment |
| 23 | Liu[23] | 2022 | Patients with positive serum hepatitis B surface antigen or positive serum HBV–DNA |
| 24 | Yau[24] | 2022 | Patients with resolved hepatitis B virus (HBV) infection (as evidenced by detectable HBV surface antibody, detectable HBV core antibody, undetectable HBV DNA, and undetectable HBV surface antigen) were eligible.  Patients with chronic HBV infection (as evidenced by detectable HBV surface antigen or HBV DNA) were eligible if they were receiving antiviral therapy and had a viral load <100 IU/mL at screening. Patients with active or resolved hepatitis C virus (HCV) infection (as evidenced by detectable HCV RNA or antibody) and who did not require antiviral therapy were eligible.  Active coinfection with HBV and HCV, or HBV and hepatitis D virus, was not permitted. |

**1.****4 Table S4:** **Risk of bias assessment.**

| **Risk of bias assessment of non-randomised intervention studies (ROBINS I)** | | | | | | | | | | | | | | | | | | | | | | | | | | | | | | | | | | | |
| --- | --- | --- | --- | --- | --- | --- | --- | --- | --- | --- | --- | --- | --- | --- | --- | --- | --- | --- | --- | --- | --- | --- | --- | --- | --- | --- | --- | --- | --- | --- | --- | --- | --- | --- | --- |
|  | | | | Bias due to confounding | | | | Bias in selection of participants into the study | | | | Bias in classification of interventions | | | | | Bias due to deviations from intended interventions | | | | Bias due to missing data | | | | Bias in measurement of outcomes | | | | Bias in selection of the reported results | | | | | Overall bias | |
| El-Khoueiry, 2017 | | | | **M** | | | | **L** | | | | **M** | | | | | **L** | | | | **L** | | | | **M** | | | | **L** | | | | | **M** | |
| Kelley, 2022 | | | | **M** | | | | **M** | | | | **L** | | | | | **M** | | | | **?** | | | | **?** | | | | **M** | | | | | **M** | |
| Lu, 2017 | | | | **M** | | | | **M** | | | | **L** | | | | | **?** | | | | **?** | | | | **M** | | | | **M** | | | | | **M** | |
| Sangro, 2013 | | | | **L** | | | | **L** | | | | **L** | | | | | **M** | | | | **M** | | | | **M** | | | | **M** | | | | | **M** | |
| Zhu, 2018 | | | | **L** | | | | **L** | | | | **M** | | | | | **L** | | | | **L** | | | | **M** | | | | **L** | | | | | **M** | |
| **Risk of bias assessment of randomised studies (ROB 2.0)** | | | | | | | | | | | | | | | | | | | | | | | | | | | | | | | | | | | |
|  | | | | Risk of bias arising from the randomization process | | | | | | Bias due to deviations from the intended interventions | | | | | Missing outcome data | | | | | | Bias in the measurement of the outcome | | | | | Bias in the selection of the reported result | | | | | | Overall bias | | | |
| Yau, 2020 | | | | **L** | | | | | | **M** | | | | | **L** | | | | | | **L** | | | | | **L** | | | | | | **M** | | | |
| Yau, 2022 | | | | **L** | | | | | | **M** | | | | | **L** | | | | | | **L** | | | | | **L** | | | | | | **M** | | | |
| **Risk of bias assessment of Observational study (STROBE)** | | | | | | | | | | | | | | | | | | | | | | | | | | | | | | | | | | | |
| Author-year  /Term | 1 | 2 | 3 | | 4 | 5 | 6 | | 7 | | 8 | | 9 | 10 | | 11 | | 12 | 13 | 14 | | 15 | 16 | 17 | | | 18 | 19 | | 20 | 21 | | 22 | | Total/Percentage  (%) |
| Cheng  2022 | 1 | 1 | 1 | | 1 | 1 | 1 | | 1 | | 1 | | 0 | 0 | | 1 | | 0 | 0 | 1 | | 1 | 0 | 0 | | | 1 | 1 | | 1 | 1 | | 1 | | **72.73%** |
| Himmelsbach  2022 | 1 | 1 | 1 | | 1 | 1 | 1 | | 0 | | 1 | | 0 | 0 | | 1 | | 0 | 0 | 1 | | 1 | 0 | 0 | | | 1 | 1 | | 1 | 1 | | 1 | | **68.18%** |
| Pan  2022 | 1 | 1 | 1 | | 1 | 1 | 1 | | 1 | | 1 | | 0 | 0 | | 1 | | 0 | 0 | 1 | | 1 | 0 | 1 | | | 1 | 1 | | 1 | 1 | | 1 | | **77.27%** |
| Pertejo-Fernandez2020 | 1 | 1 | 1 | | 0 | 1 | 1 | | 0 | | 0 | | 0 | 0 | | 0 | | 0 | 1 | 1 | | 1 | 0 | 0 | | | 1 | 1 | | 1 | 1 | | 1 | | **59.09%** |
| Shah  2019 | 1 | 0 | 1 | | 0 | 1 | 1 | | 0 | | 0 | | 0 | 0 | | 0 | | 0 | 1 | 1 | | 1 | 0 | 0 | | | 1 | 0 | | 1 | 1 | | 0 | | **45.45%** |
| Tsimafeyeu  2020 | 1 | 1 | 1 | | 1 | 1 | 1 | | 1 | | 1 | | 0 | 0 | | 1 | | 1 | 0 | 1 | | 1 | 0 | 0 | | | 1 | 1 | | 1 | 1 | | 1 | | **77.27%** |
| Xu  2021 | 1 | 1 | 1 | | 1 | 1 | 1 | | 0 | | 1 | | 0 | 0 | | 0 | | 0 | 0 | 1 | | 1 | 0 | 0 | | | 1 | 1 | | 1 | 1 | | 1 | | **63.64%** |
| Zhang  2021 | 1 | 1 | 1 | | 1 | 1 | 1 | | 1 | | 0 | | 0 | 0 | | 1 | | 0 | 1 | 1 | | 1 | 0 | 0 | | | 1 | 1 | | 1 | 1 | | 1 | | **72.73%** |
| Zhong  2021 | 1 | 1 | 1 | | 0 | 1 | 1 | | 1 | | 0 | | 1 | 0 | | 1 | | 1 | 0 | 1 | | 1 | 0 | 0 | | | 1 | 1 | | 1 | 1 | | 1 | | **72.73%** |
| Yuan  2021 | 1 | 1 | 1 | | 1 | 1 | 1 | | 1 | | 1 | | 0 | 0 | | 1 | | 0 | 1 | 1 | | 1 | 0 | 1 | | | 1 | 1 | | 1 | 1 | | 1 | | **81.82%** |
| Chen J  2020 | 1 | 1 | 1 | | 1 | 1 | 1 | | 1 | | 1 | | 0 | 0 | | 0 | | 1 | 1 | 1 | | 1 | 0 | 0 | | | 1 | 1 | | 1 | 1 | | 1 | | **77.27%** |
| Chen C  2020 | 1 | 1 | 1 | | 1 | 1 | 1 | | 1 | | 0 | | 0 | 0 | | 0 | | 1 | 1 | 1 | | 1 | 0 | 1 | | | 1 | 1 | | 1 | 1 | | 1 | | **77.27%** |
| Kothapalli2018 | 1 | 1 | 1 | | 1 | 0 | 0 | | 1 | | 1 | | 0 | 0 | | 0 | | 0 | 1 | 1 | | 1 | 1 | 0 | | | 1 | 1 | | 1 | 1 | | 1 | | **68.18%** |
| Tio  2018 | 1 | 1 | 1 | | 1 | 1 | 1 | | 1 | | 0 | | 0 | 0 | | 0 | | 0 | 1 | 1 | | 1 | 0 | 0 | | | 1 | 1 | | 1 | 1 | | 1 | | **68.18%** |
| Ravi  2014 | 1 | 1 | 1 | | 0 | 1 | 0 | | 0 | | 0 | | 0 | 0 | | 0 | | 0 | 1 | 1 | | 1 | 0 | 0 | | | 1 | 1 | | 1 | 1 | | 1 | | **54.55%** |
| Hu2022 | 1 | 1 | 1 | | 1 | 1 | 1 | | 1 | | 1 | | 0 | 0 | | 0 | | 1 | 0 | 1 | | 1 | 0 | 1 | | | 1 | 1 | | 1 | 1 | | 1 | | **77.27%** |
| Liu2022 | 1 | 1 | 1 | | 1 | 1 | 1 | | 1 | | 1 | | 0 | 0 | | 0 | | 1 | 0 | 1 | | 1 | 0 | 0 | | | 1 | 1 | | 1 | 1 | | 1 | | **72.73%** |

Risk of bias assessed by the ROBINS-I tool is classified per category as either **L** (low), **M** (moderate), **S** (serious), **C** (critical) or **?** (no information). Risk of bias assessed by the ROB tool is classified per category as either **L** (low), **M** (some concerns), **H** (high) or **?** (no information). Risk of bias assessed by the STROBE is classified 22 items as 1(yes), 0(no). According to the percentage of "1", it is divided into **high risk** (0%-25%), **medium to high risk** (25% -50%), **low to medium risk** (50%-75%) and **low risk** (75%-100%).

**2. Supplementary Figures**

**2.1** **Figure S1: Subgroup analysis of (A)ORR, (B)DCR, (C)mPFS, (D)mOS.**


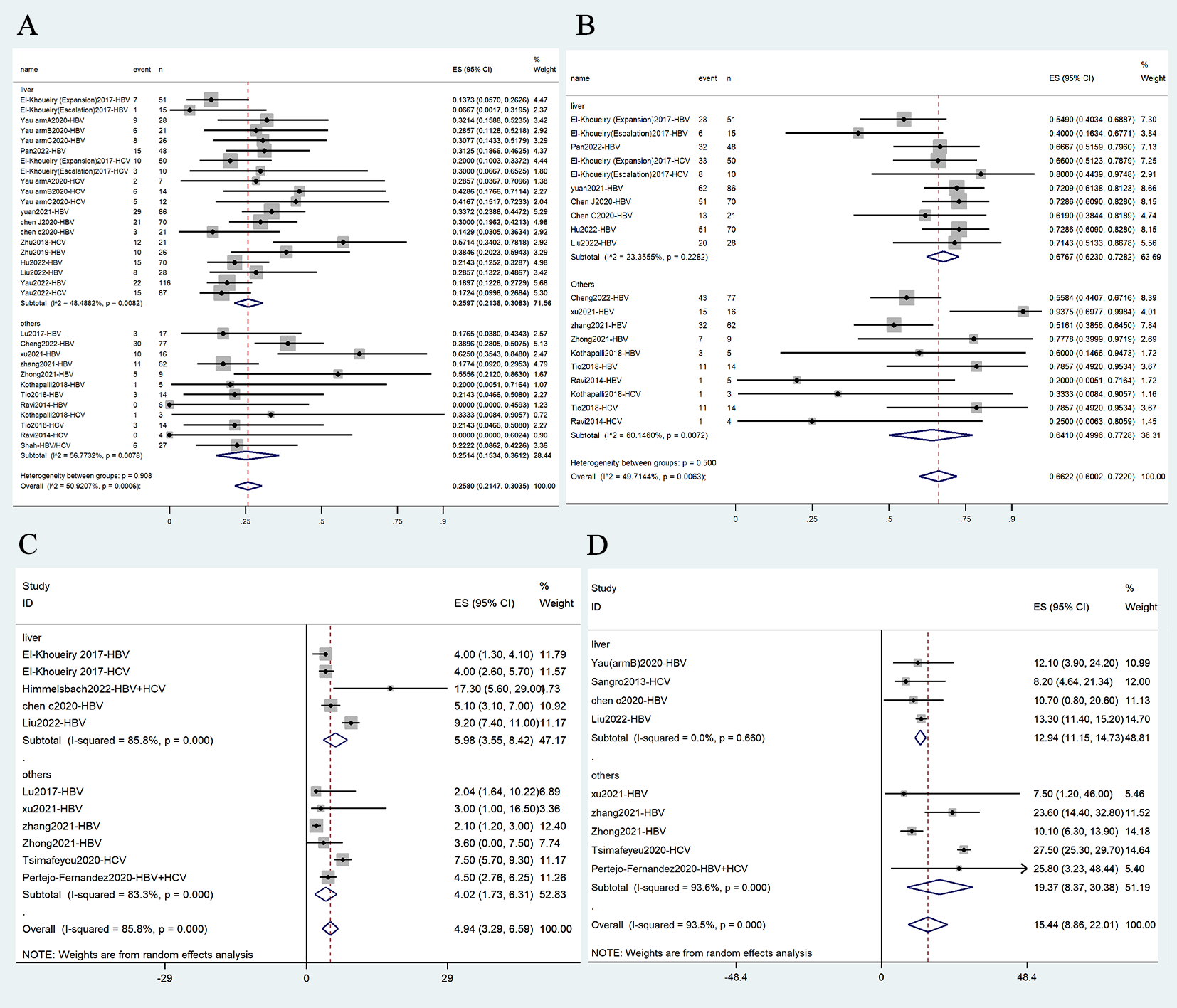


**2.2** **Figure S2: Subgroup analysis of(A) all grades irAEs, (B)grades3-4 irAEs.**


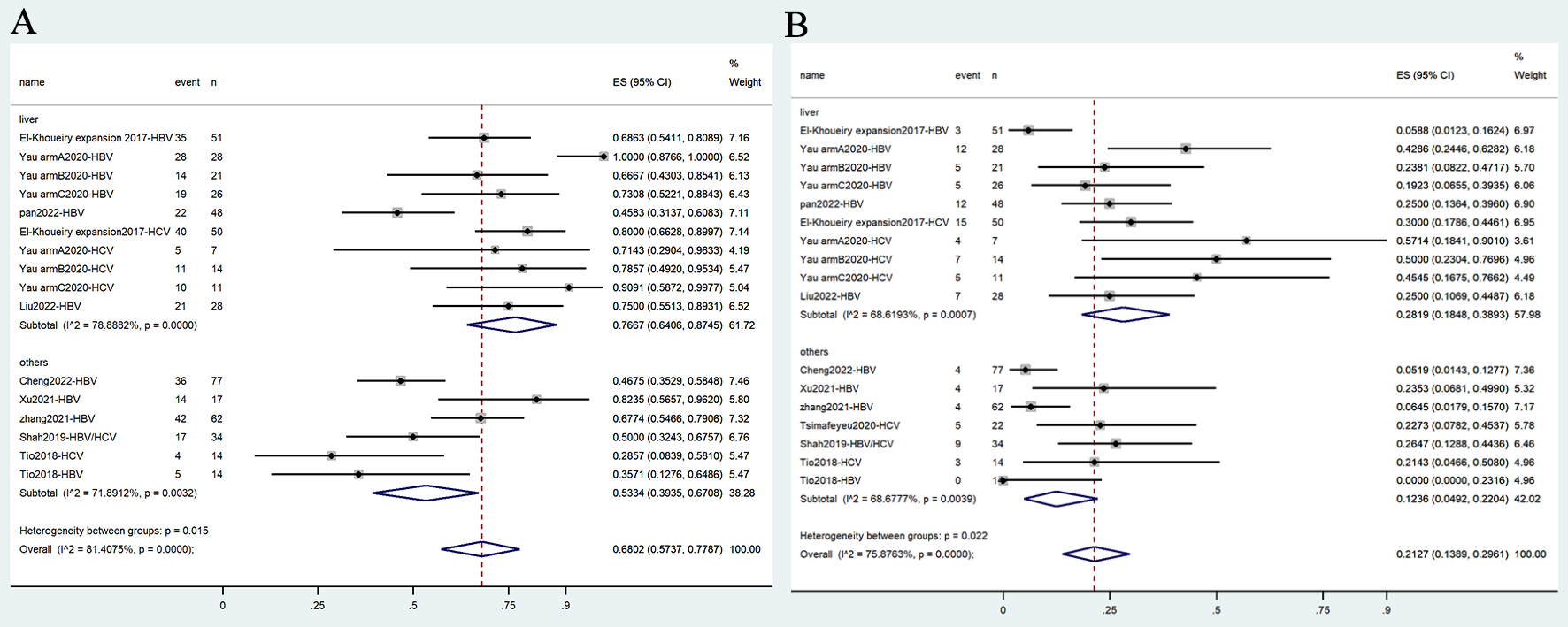


**2.3 Figure S3: The pooled incidence rate of AST, and ALT abnormalities. (A)all grades incidence rate of AST;(B) grades3-4 incidence rate of AST; (C) all grades incidence rate of ALT; (D) grades3-4 incidence rate of ALT.**


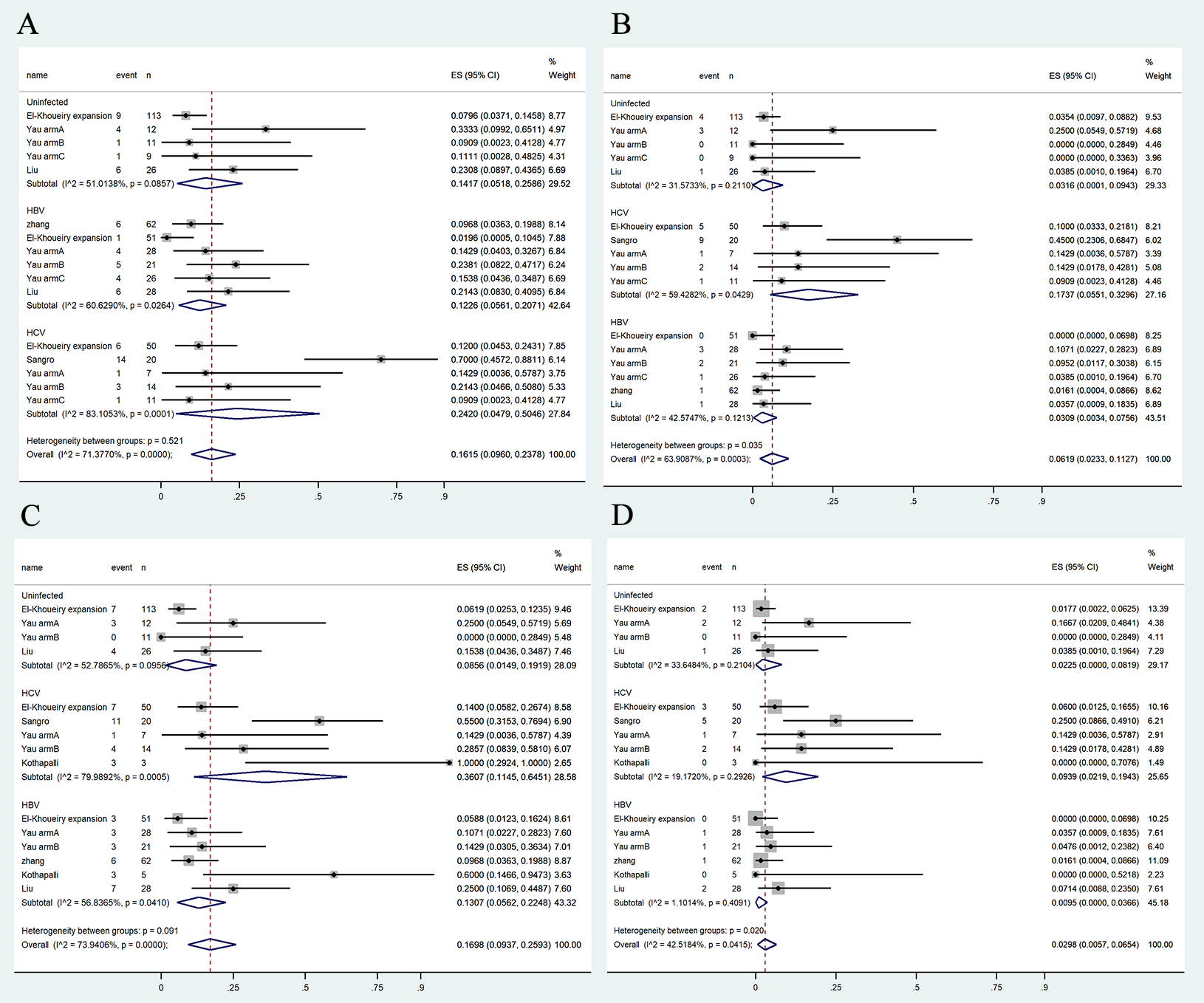


**2.4 Figure S4:** **The subgroup analysis of ICIs .(A)ORR; (B) DCR; (C) mOS; (D) mPFS; (E) all grades irAEs; (F) grades3-4 irAEs; (G) all grades incidence rate of ALT; (H) grades3-4 incidence rate of ALT ; (I) all grades incidence rate of AST; (J) grades3-4 incidence rate of AST.**


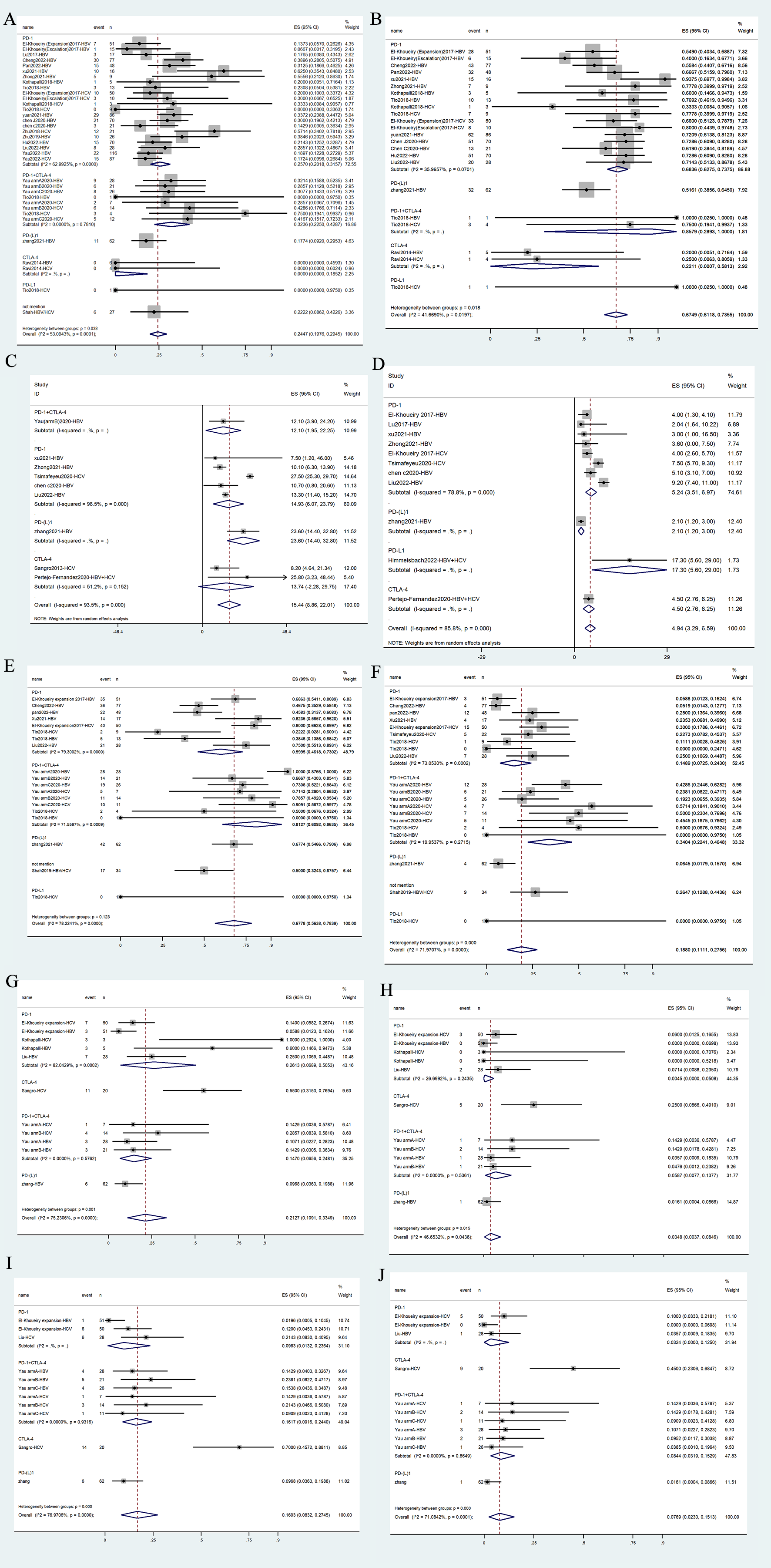


**2.5 Figure S5:** **The sensitivity analysis. (A)ORR of patients with HBV; (B) ORR of patients with HCV; (C) DCR of patients with HBV; (D) DCR of patients with HCV;(E) mOS of patients with HBV;(F) mPFS of patients with HBV;(G) all grades irAEs of patients with HBV; (H) all grades irAEs of patients with HCV; (I) grades3-4 irAEs of patients with HBV; (J) grades3-4 irAEs of patients with HCV.**

**
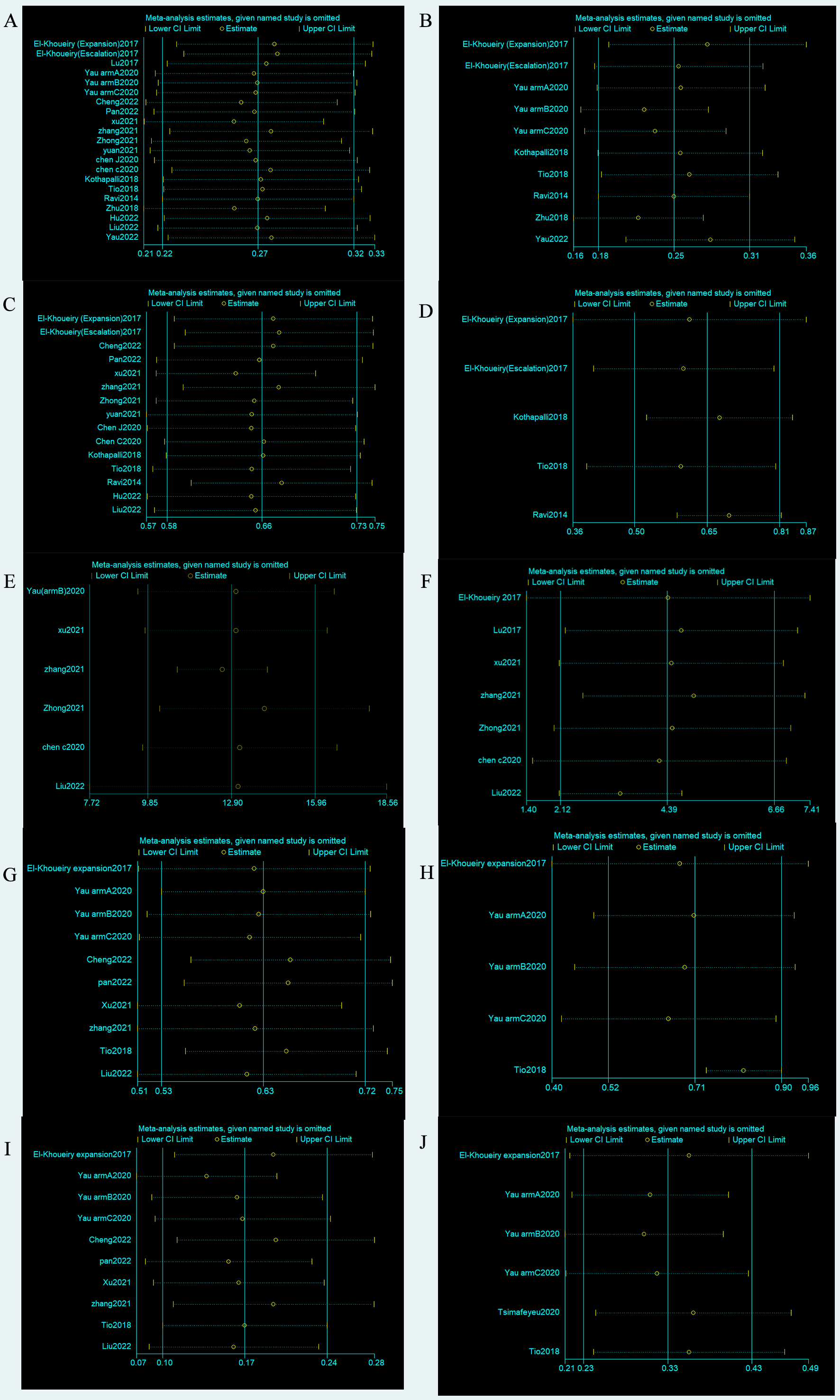
**

[1] A.B. El-Khoueiry, B. Sangro, T. Yau, et al., Nivolumab in patients with advanced hepatocellular carcinoma (CheckMate 040): an open-label, non-comparative, phase 1/2 dose escalation and expansion trial, Lancet 2017;**(no pagination)**(

[2] R.K. Kelley, P.M. Bracci, B. Keenan, et al., Pembrolizumab (PEM) plus granulocyte macrophage colony stimulating factor (GM-CSF) in advanced biliary cancers (ABC): Final outcomes of a phase 2 trial, Journal of Clinical Oncology 2022;**40**(4 SUPPL):

[3] S. Lu, L. Zhang, Y. Cheng, et al., Checkmate 870: An open-label safety study of nivolumab in previously treated patients with non-small cell lung cancer in ASIA, Journal of Thoracic Oncology 2017;**12**(11): S1976.

[4] B. Sangro, C. Gomez-Martin, M. De La Mata, et al., A clinical trial of CTLA-4 blockade with tremelimumab in patients with hepatocellular carcinoma and chronic hepatitis C, Journal of Hepatology 2013;**59**(1): 81-88.

[5] T. Yau, Y.K. Kang, T.Y. Kim, et al., Efficacy and Safety of Nivolumab Plus Ipilimumab in Patients With Advanced Hepatocellular Carcinoma Previously Treated With Sorafenib The CheckMate 040 Randomized Clinical Trial, JAMA ONCOLOGY 2020;**6**(11):

[6] A.X. Zhu, R.S. Finn, J. Edeline, et al., Pembrolizumab in patients with advanced hepatocellular carcinoma previously treated with sorafenib (KEYNOTE-224): a non-randomised, open-label phase 2 trial, LANCET ONCOLOGY 2018;**19**(7): 940-952.

[7] Y.K. Cheng, P. Chen, D.W. Chen, et al., Comparative Safety, Efficacy and Survival Outcome of Anti-PD-1 Immunotherapy in Colorectal Cancer Patients With vs Without Hepatitis B Virus Infection: A Multicenter Cohort Study, Clin Transl Gastroenterol 2022;**13**(5): e00475.

[8] V. Himmelsbach, M. Pinter, B. Scheiner, et al., Efficacy and Safety of Atezolizumab and Bevacizumab in the Real-World Treatment of Advanced Hepatocellular Carcinoma: Experience from Four Tertiary Centers, CANCERS 2022;**14**(7):

[9] S. Pan, Y. Yu, S. Wang, et al., Correlation of HBV DNA and Hepatitis B Surface Antigen Levels With Tumor Response, Liver Function and Immunological Indicators in Liver Cancer Patients With HBV Infection Undergoing PD-1 Inhibition Combinational Therapy, Front Immunol 2022;**13**(892618.

[10] A. Pertejo-Fernandez, B. Ricciuti, S.P. Hammond, et al., Safety and efficacy of immune checkpoint inhibitors in patients with non-small cell lung cancer and hepatitis B or hepatitis C infection, Lung Cancer 2020;**145**(181-185.

[11] N.J. Shah, G. Al-Shbool, M. Blackburn, et al., Safety and efficacy of immune checkpoint inhibitors (ICIs) in patients with HIV, hepatitis B, or hepatitis C viral infections, Cancer Research 2019;**79**(13):

[12] I. Tsimafeyeu, R. Gafanov, S. Protsenko, et al., Nivolumab in patients with metastatic renal cell carcinoma and chronic hepatitis C virus infection, Cancer Immunology, Immunotherapy 2020;**69**(6): 983-988.

[13] F. Xu, Z. Zeng, B. Yan, et al., Safety and efficacy of anti-PD-1 inhibitors in Chinese patients with advanced lung cancer and hepatitis B virus infection: a retrospective single-center study, Transl Lung Cancer Res 2021;**10**(4): 1819-1828.

[14] X.Y. Zhang, D. Tian, Y. Chen, et al., Association of hepatitis B virus infection status with outcomes of non-small cell lung cancer patients undergoing anti-PD-1/PD-L1 therapy, TRANSLATIONAL LUNG CANCER RESEARCH 2021;**10**(7): 3191-3202.

[15] L.T. Zhong, P.S. Zhong, H.F. Liu, et al., Hepatitis B virus infection does not affect the clinical outcome of anti-programmed death receptor-1 therapy in advanced solid malignancies Real-world evidence from a retrospective study using propensity score matching, MEDICINE 2021;**100**(49):

[16] G.S. Yuan, R. Li, Q. Li, et al., Interaction between hepatitis B virus infection and the efficacy of camrelizumab in combination with apatinib therapy in patients with hepatocellular carcinoma: a multicenter retrospective cohort study, ANNALS OF TRANSLATIONAL MEDICINE 2021;**9**(18):

[17] J. Chen, X. Hu, Q. Li, et al., Effectiveness and safety of toripalimab, camrelizumab, and sintilimab in a real-world cohort of hepatitis B virus associated hepatocellular carcinoma patients, Annals of Translational Medicine 2020;**8**(18):

[18] C. Chen, L. An, Y. Cheng, et al., Clinical Outcomes and Prognosis Factors of Nivolumab Plus Chemotherapy or Multitarget Tyrosine Kinase Inhibitor in Multi-Line Therapy for Recurrent Hepatitis B Virus-Related Hepatocellular Carcinoma: A Retrospective Analysis, Frontiers in Oncology 2020;**10**(

[19] A. Kothapalli, M.A. Khattak, Safety and efficacy of anti-PD-1 therapy for metastatic melanoma and non-small-cell lung cancer in patients with viral hepatitis: A case series, Melanoma Research 2018;**28**(2): 155-158.

[20] M. Tio, R. Rai, O.M. Ezeoke, et al., Anti-PD-1/PD-L1 immunotherapy in patients with solid organ transplant, HIV or hepatitis B/C infection, European Journal of Cancer 2018;**104**(137-144.

[21] S. Ravi, K. Spencer, M. Ruisi, et al., Ipilimumab administration for advanced melanoma in patients with pre-existing Hepatitis B or C infection: A multicenter, retrospective case series, Journal for ImmunoTherapy of Cancer 2014;**2**(1):

[22] X. Hu, R. Li, Q. Li, et al., Interaction between baseline HBV loads and the prognosis of patients with HCC receiving anti-PD-1 in combination with antiangiogenic therapy undergoing concurrent TAF prophylaxis, BMC Infectious Diseases 2022;**22**(1): 614.

[23] H. Liu, X. Qin, Z. Xu, et al., Comparison of effectiveness and safety of camrelizumab between HBV-related and non-B, non-C hepatocellular carcinoma: A retrospective study in China, Frontiers In Genetics 2022;**13**(1000448.

[24] T. Yau, J.W. Park, R.S. Finn, et al., Nivolumab versus sorafenib in advanced hepatocellular carcinoma (CheckMate 459): a randomised, multicentre, open-label, phase 3 trial, LANCET ONCOLOGY 2022;**23**(1): 77-90.
